# Broiler Responses to a Phytogenic Nutritional Additive Under Different Dietary Nutrient Matrix Credit Strategies

**DOI:** 10.3390/ani16152381

**Published:** 2026-08-03

**Authors:** Ze Wang, Jianjun Wang, Yong Yu, Bingjian Huang, Lilan Xu, Ya Lu, Guangzhan Ma, Yuan Yue, Kaichen Zheng, Xianlei Li, Bingkun Zhang

**Affiliations:** 1 State Key Laboratory of Animal Nutrition and Feeding, Department of Animal Nutrition and Feed Science, College of Animal Science and Technology, China Agricultural University, No. 2 Yuanmingyuan West Road, Haidian District, Beijing 100193, China; wangze@cau.edu.cn (Z.W.); wangj2022@126.com (J.W.); 17853148163@163.com (B.H.); s20243040845@cau.edu.cn (Y.L.); 17305241961@163.com (G.M.); yyuan552990@163.com (Y.Y.); 13390633998@163.com (K.Z.); 2 Cargill Investments (China), Ltd., 10F, One ICC, Shanghai International Commerce Center, 999 Huaihai Road (M), Xuhui District, Shanghai 200031, China; yong_yu@cargill.com (Y.Y.); 18262602925@163.com (L.X.); 3 Beijing SinoAgri Uplus Biotech Co., Ltd., No. 10 Fengrun East Road, Haidian District, Beijing 100094, China; 18810918693@163.com

**Keywords:** phytogenic nutritional additive, broiler, nutrient matrix, feed formulation, intestinal barrier, excreta composition

## Abstract

This study evaluated the physiological and production responses of broilers to a phytogenic nutritional additive (PNA) applied under different dietary nutrient matrix credit strategies. The PNA contained star anise essential oil, thyme essential oil, and *Quillaja saponaria* Molina stem and leaf powder, with trans-anethole, p-cymene, and thymol as the main characterized marker compounds. A total of 384 one-day-old male Arbor Acres broilers were assigned to four treatments (eight replicates of 12 birds): a basal control diet without PNA (CON) and PNA-supplemented diets using full matrix credit (PNA-FM), protein matrix credit (PNA-PM), or no matrix credit (PNA-NM). Growth performance was unaffected (*p* > 0.05), whereas all PNA-supplemented groups had higher dressed percentages and eviscerated yields than CON (*p* < 0.01). PNA-PM and PNA-NM increased the muscle pH (*p* < 0.01), and PNA-NM increased drip loss and shear force (*p* < 0.05). Intestinal responses were time-dependent: on day 14, PNA-NM increased serum FITC-d, tended to increase DAO activity, and downregulated *ZO-1* expression, while all PNA-containing treatments reduced the villus height and villus height-to-crypt depth ratio (*p* < 0.01), suggesting a transient epithelial adjustment rather than direct barrier improvement. On day 22, qualitative TEM observations suggested closer epithelial junctions in PNA-containing treatments. These results indicate that PNA-containing formulation strategies did not compromise growth performance and modified carcass traits, meat quality traits, intestinal responses, and the excreta nutrient composition. Because PNA-free full matrix and protein matrix control diets were not included, these findings should be interpreted as responses to complete dietary strategies rather than as formal PNA × nutrient matrix interactions.

## Simple Summary

Plant-based feed additives are increasingly used in commercial poultry farming as natural options to support bird health and productivity. However, farmers and feed producers need practical advice on how to best include these products in everyday feed recipes. This study tested a phytogenic nutritional additive (PNA) containing star anise essential oil, thyme essential oil, and *Quillaja saponaria* Molina stem and leaf powder in chicken diets under different feed formulation strategies. The goal was to investigate if these strategies could support chicken growth while also affecting meat quality, gut health, and nutrient loss. The results showed that growth performance was not affected by the PNA-containing formulation strategies. These strategies also influenced nutrient utilization and intestinal health, and the effects depended on the specific nutrient matrix strategy applied. Interestingly, early signs pointed to a temporary adjustment in the gut lining, which was followed by signs of stronger connections between gut cells by day 22. These findings offer useful information for feed manufacturers on how to use plant-based additives more effectively, helping to balance bird performance, meat yields, and digestive health in modern poultry nutrition.

## 1. Introduction

Phytogenic nutritional additives (PNAs) have gained widespread acceptance in broiler nutrition as functional additives. Under commercial feeding conditions, they are primarily utilized to support intestinal physiology and optimize nutrient utilization [1,2]. Furthermore, recent investigations highlight their critical potential in modulating microbial balance and overall gut health [3,4,5]. Specifically, essential oil- and phytobiotic-based products have been reported to influence growth performance, nutrient-related responses [6,7], intestinal morphology, immune responses [8,9], and barrier-related functions [10]. Despite these documented benefits, the magnitude and direction of broiler responses to these additives are not always consistent. These variations are often highly dependent on the diet composition, the specific formulation of the additive, and the environmental or health challenge status of the flock.

In commercial practice, the evaluation of functional additives is further complicated by the routine application of dietary matrix credits. Assigning a matrix credit to an additive accounts for its presumed nutrient contribution, resulting in intentional reductions in the basal formulation of energy-yielding ingredients, crude protein, standardized ileal digestible amino acids, calcium, and phosphorus. These baseline nutrient adjustments are biologically significant. For instance, altered protein and amino acid dynamics directly impact nutrient utilization and overall production efficiency in broilers [11,12], whereas shifts in calcium and non-phytate phosphorus supply heavily influence skeletal development and intestinal physiology [13]. Furthermore, studies utilizing reduced-energy or reduced-amino-acid diets demonstrate that broiler performance and carcass traits are highly sensitive to both the magnitude and type of nutrient restriction [14,15]. Consequently, the ultimate gut health and production outcomes are driven not solely by the inclusion of functional additives but by their complex interplay with the specific matrix-adjusted diet [16].

The PNA evaluated in the present study is a botanical-based feed additive derived from star anise (*Illicium verum* Hook.f.) essential oil, thyme (*Thymus vulgaris* L.) essential oil, and *Quillaja saponaria* Molina stem and leaf powder. Star anise and thyme are aromatic plants that are widely used as sources of essential oils in animal nutrition due to their bioactive compounds, including trans-anethole and thymol, which have been associated with antimicrobial, antioxidant, and digestive-supporting activities. Previous studies have indicated that star anise-derived compounds can improve nutrient utilization and intestinal barrier function in broilers [17,18], while thyme-derived phytochemicals may contribute to the modulation of intestinal microbial balance and inflammatory responses [19]. In addition, quillaja saponins have attracted attention as natural feed additives because of their potential effects on intestinal health, nutrient absorption, and immune regulation. However, biological responses to botanical mixtures may depend on the composition, inclusion level, and dietary context, and the combined effects of these plant-derived components under different nutrient matrix formulation strategies remain insufficiently understood. Similarly, thymol exhibits potent antimicrobial and antioxidant capacities that promote intestinal health in poultry [20,21]. Crucially, rather than acting in isolation, these volatile components often interact synergistically. For example, p-cymene is known to potentiate phenolic monoterpenes via membrane-level effects [22], leading to enhanced antibacterial activity [23,24]. Furthermore, the broader spice-derived and phytogenic compounds (such as chlorogenic acid and esterified derivatives) provide additional regulatory support by modulating antioxidant, inflammatory, and microbial responses [25,26]. Because this PNA functions as a complex, multi-targeted network rather than a single molecule, its overall efficacy in vivo may be particularly sensitive to the nutritional baseline provided by different matrix credit formulations.

Currently, limited information is available regarding broiler responses to PNAs when applied under different nutrient matrix credit strategies. In commercial poultry production, practical formulation strategies often compare a standard control diet with additive-containing diets formulated using different matrix credits, rather than using a complete factorial arrangement of additive and matrix levels. Because PNA-free full matrix and protein matrix control diets were not included, the present design did not allow the formal testing of independent PNA effects, independent nutrient matrix effects, or PNA × nutrient matrix interactions. The present study was therefore conducted to compare a control diet without PNA with three complete PNA-containing dietary formulation strategies: full nutrient matrix credit, protein matrix credit, and no matrix credit. The objective was to evaluate treatment-dependent responses in growth performance, carcass traits, meat quality, intestinal barrier-related indices, ileal morphology, epithelial ultrastructure, and excreta nutrient composition. Because corresponding PNA-free matrix control diets were not included, differences among PNA-containing strategies were interpreted as matrix-dependent response patterns rather than formal statistical interactions.

## 2. Materials and Methods

### 2.1. Phytogenic Nutritional Additive

A phytogenic nutritional additive (PNA; Delacon Biotechnik GmbH, Engerwitzdorf, Austria), consisting mainly of star anise essential oil, thyme essential oil, and *Quillaja saponaria* Molina stem and leaf powder, was used in this study. The characterized essential oil fraction contained trans-anethole (25 mg/g), p-cymene (4 mg/g), and thymol (2 mg/g) as the main active components. The inclusion level was fixed at 0.015% (150 mg/kg) across all feeding phases.

### 2.2. Experimental Design and Diets

A total of 384 one-day-old male Arbor Acres broilers with an initial body weight of 42.16 ± 0.60 g were obtained from Shandong Dingli Agricultural and Animal Husbandry Technology Co., Ltd. (Yantai, China). Birds were randomly allocated to four dietary treatments, with eight replicate cages per treatment and 12 birds per cage. The treatments consisted of a control diet without PNA (CON), PNA with a full nutrient matrix credit (PNA-FM), PNA with a protein matrix credit (PNA-PM), and PNA without a matrix credit (PNA-NM). In this study, matrix credit refers to a pre-assigned nutrient value attributed to the additive during diet formulation. When a matrix credit was applied, selected nutrients or ingredients in the basal formulation were adjusted according to the expected nutritional contribution or functional effect of the additive. Thus, the matrix credit strategies represented practical feed formulation approaches rather than independent nutrient restriction treatments.

The PNA-FM diet represented a full-nutrient-matrix credit strategy. Compared with CON, this strategy applied the broader nutrient matrix attributed to the PNA and reduced the ether extract by approximately 8.0 to 9.3%, calcium and phosphorus by approximately 3%, and selected indispensable amino acids, such as lysine and methionine, by approximately 1.9% and 3.9%, respectively, while maintaining crude protein and metabolizable energy at similar levels. The PNA-PM diet represented a protein-matrix-credit strategy, in which the crude protein and standardized ileal digestible amino acid balance were adjusted, whereas the broader full matrix credit for ether extract and minerals was not fully applied. The PNA-NM diet represented a no-matrix-credit strategy, in which the PNA was added at the same inclusion level to the control nutrient specification without nutrient matrix deductions.

The matrix credit adjustments were intentionally conservative and were designed to reflect practical formulation margins rather than severe nutrient restriction. Because the study did not include PNA-free full matrix or protein matrix diets, treatment differences in PNA-FM and PNA-PM were interpreted as responses associated with complete PNA-containing formulation strategies, not as independent effects of the PNA alone or as formal PNA × nutrient matrix interactions. The basal diets followed current broiler nutritional recommendations and were formulated using raw materials from the Zhuozhou Experimental Base of China Agricultural University (Zhuozhou, China) with technical optimization from Cargill. The ingredient compositions and nutrient levels of the basal diets are shown in Table 1. Birds had ad libitum access to feed and water during the 43-day experiment, and the temperature and lighting were managed according to the Arbor Acres broiler management guide.

### 2.3. Diet Preparation and Chemical Analysis

The corn–soybean meal basal diet was used in all feeding phases. Ingredients and additives were weighed according to the diet formulas and premixed stepwise before final mixing in a horizontal ribbon-blade mixer and pelleting. Diet samples were ground and analyzed in duplicate. Dry matter, crude protein, ether extract, and ash were determined according to AOAC procedures [27]. Metabolizable energy, total calcium, total phosphorus, and tryptophan were calculated based on ingredient nutrient composition tables and formulation specifications. Nutrient levels are expressed on an as-fed basis.

### 2.4. Growth Performance

Body weight (BW), average daily gain (ADG), average daily feed intake (ADFI), and the feed conversion ratio (FCR) were recorded on a cage basis for each feeding phase and for the overall experimental period. Mortality and culled birds were recorded daily for each replicate cage.

### 2.5. Sample Collection

At day 14, six birds per treatment were sampled, with one bird selected from each of six different replicate cages among the eight replicate cages per treatment. Three hours before sampling, birds received 4.16 mg fluorescein isothiocyanate–dextran (FITC-d)/kg body weight by oral gavage. Birds were electrically stunned and euthanized by exsanguination. Blood was collected from the jugular vein into additive-free dry vacuum tubes, allowed to clot for 3 h at room temperature, and centrifuged at 1500× *g* for 15 min. Serum was stored at −80 °C for the analysis of FITC-d, D-lactic acid (D-LA), and diamine oxidase (DAO). Ileal tissue and mucosal samples were collected between Meckel’s diverticulum and the ileocecal junction, frozen in liquid nitrogen, and stored at −80 °C.

At day 22, five birds per treatment were sampled from five different replicate cages among the eight replicate cages per treatment, with one bird selected per cage; these birds had not been previously sampled at day 14. Ileal samples were collected for histomorphology and transmission electron microscopy (TEM). Samples for TEM were cut into approximately 1 to 2 mm^3^ sections and immediately immersed in 10 volumes of 2.5% glutaraldehyde at 4 °C. During collection, forceps compression was avoided to preserve the intestinal morphology.

At day 42, one bird was sampled from each available replicate cage among the eight replicate cages per treatment for breast meat measurements. After slaughter, the breast was exposed and photographed for white striping evaluation. A 1.5 cm × 4.0 cm strip from the center of the right pectoralis major muscle was used for pH measurement. Two additional 1 cm × 1 cm samples were collected from the same region; one sample was frozen in liquid nitrogen and stored at −80 °C for the analysis of malondialdehyde (MDA), glutathione (GSH), and total antioxidant capacity (T-AOC), and the other was fixed in 10 volumes of paraformaldehyde for the determination of the collagen volume fraction (CVF) and muscle fiber diameter (MFD). The left pectoralis major muscle was stored at −20 °C for cooking loss analysis.

At day 43, two birds were sampled from each available replicate cage among the eight replicate cages per treatment for carcass measurements. The dressed percentage (DP), half-eviscerated yield with giblets (HEP), eviscerated yield (EP), abdominal fat yield (AEP), breast muscle yield (BMP), and leg muscle yield (LMP) were calculated according to standard slaughter procedures. For carcass analysis, values from the two birds were averaged within the cage before statistical analysis. Excreta was collected from six replicate cages per treatment at days 14 and 28 for the determination of moisture, crude protein, ether extract, gross energy, ash, crude fiber, calcium, and phosphorus. Moisture was additionally measured at day 42.

### 2.6. Clinical Observations and Lesion Evaluation

Throughout the 42-day experimental period, birds were monitored daily for general health status and clinical signs of illness. Scheduled gross lesion evaluations were conducted from day 14 to day 42, with a total of 21 clinically healthy birds examined per treatment over this period. The evaluated gross lesion indicators included oral lesions (ML), intestinal hemorrhage (IH), excessive intestinal mucus (MC), excessive intestinal water content (WC), intestinal epithelial cell shedding (CS), intestinal hyperemia (HY), poor intestinal elasticity (IT), gizzard erosion (GIZ), and cecal gas bubbles, used as an indicator of microbial imbalance (CFM). Binary indicators were scored as 0 = absent and 1 = present, whereas intestinal hemorrhage and gizzard erosion were graded on a 0 to 3 scale, with 0 indicating absence and higher scores indicating greater lesion severity. For lesion incidence analysis, birds with a score greater than 0 for a given indicator were considered lesion-positive. The lesion incidence was calculated as the number of lesion-positive birds divided by the number of birds examined within each treatment and expressed as a percentage. The average lesion incidence was calculated as the mean percentage of lesion-positive birds across the recorded gross lesion indicators from day 14 to day 42.

### 2.7. Meat Quality and Muscle Antioxidant Indices

Cooking loss was determined after thawing the left pectoralis major muscle samples at 4 °C, using a procedure adapted from Gál et al. [28]. Samples were weighed (W1), sealed in polyethylene bags, and cooked in a water bath at 85 °C until the internal center temperature reached 70 °C. After cooling to ambient temperature in tap water and storage at 4 °C for 24 h, samples were blotted dry and reweighed (W2). Cooking loss was calculated as 100 × (W1 − W2)/W1.

For muscle antioxidant indices, approximately 0.1 g of muscle tissue was homogenized in normal saline to prepare a ten-fold dilution and centrifuged at 3500× *g* for 15 min at 4 °C. The protein concentration was determined using a BCA Protein Assay Kit (P0012, Beyotime Biotechnology, Nantong, Jiangsu, China). MDA, GSH, and T-AOC were quantified using commercial kits (Nanjing Jiancheng Bioengineering Institute, Nanjing, Jiangsu, China), according to the manufacturers’ instructions. MDA was measured at 532 nm, whereas GSH and T-AOC were measured at 405 nm using a microplate reader (Epoch 2, BioTek Instruments, Winooski, VT, USA). Results were normalized to the protein concentration.

For Masson staining, muscle tissue fixed in 4% paraformaldehyde for more than 24 h was stained using a Masson Trichrome Stain Kit (Solarbio G1340, Beijing, China). Images were collected using an SQD-40P slide scanning system (Shenzhen Shengqiang Technology Co., Ltd., Shenzhen, China). CVF was calculated as the ratio of the blue-stained collagen fiber area to the total muscle cross-sectional area. MFD was calculated by averaging the long and short axes of observed muscle fibers. Images were analyzed using ImageJ (version 1.53k, National Institutes of Health, Bethesda, MD, USA).

The white striping score was evaluated using the classification scheme described by Kuttappan et al. [29], where 0 = normal, 1 = moderate, 2 = severe, and 3 = extreme. All evaluations were conducted under standardized conditions by trained observers blinded to treatment.

### 2.8. Transmission Electron Microscopy

Ileal samples collected at day 22 were fixed in 2.5% glutaraldehyde and stored at 4 °C until further processing, following the approaches used for intestinal ultrastructure assessment in poultry [30,31]. Samples were washed three times with 0.1 M phosphate buffer, secondarily fixed with 1% osmium tetroxide, dehydrated through a graded ethanol series, embedded in epoxy resin, sectioned at approximately 50 nm, double-stained with uranyl acetate and lead citrate, and examined by transmission electron microscopy. Ultrastructural characteristics, including the integrity of tight junctions, zonula adherens, and desmosomes, were evaluated qualitatively by an independent observer blinded to the dietary treatments.

### 2.9. FITC-d and Intestinal Barrier-Related Assays

The serum FITC-d concentration was used as a relative indicator of intestinal permeability at day 14, consistent with the use of paracellular permeability markers in gut barrier research and poultry studies [32,33,34]. Serum samples and standards were analyzed in triplicate, and serum from chickens not administered FITC-d was used as the blank control. Fluorescence was measured using a Synergy MX microplate reader (BioTek Instruments, Inc., Winooski, VT, USA) at an excitation wavelength of 485 nm and an emission wavelength of 530 nm. D-Lactic acid and DAO were quantified using commercial colorimetric assay kits (Elabscience Biotechnology Co., Ltd., Wuhan, China).

For mucosal analysis, approximately 0.1 g of ileal mucosa was homogenized in a ten-fold volume of normal saline and centrifuged at 10,000× *g* for 10 min at 4 °C. Supernatants were used to determine secretory immunoglobulin A (sIgA) and cyclooxygenase-2 (COX-2) using ELISA kits (Shanghai Enzyme-Linked Biotechnology Co., Ltd., Shanghai, China).

### 2.10. Gene Expression in the Ileum

Total RNA was extracted from ileal tissue using Trizol reagent (Vazyme Biotech Co., Ltd., Nanjing, China), according to the manufacturer’s protocol. Total RNA (1 μg) was reverse-transcribed into cDNA using HiScript III All-in-One RT SuperMix Perfect (Vazyme Biotech Co., Ltd.), and the final cDNA was diluted with nuclease-free water before qPCR analysis. The qPCR reaction mixture contained 2× Taq Pro Universal SYBR qPCR Master Mix (Vazyme Biotech Co., Ltd., Nanjing, China), forward and reverse primers, nuclease-free water, and cDNA. Reactions were performed on a 7500 ABI Prism Sequence Detection System (Applied Biosystems, Foster City, CA, USA) under the following conditions: 95 °C for 30 s, followed by 40 cycles of 95 °C for 10 s and 60 °C for 30 s.

Relative gene expression was normalized to β-actin, the endogenous reference gene listed in Table A1, and calculated using the 2^−ΔΔCt^ method, with the CON group used as the calibrator [35]. Primer sequences and GenBank accession numbers are provided in Table A1. Dissociation curves were generated to verify amplification specificity.

### 2.11. Statistical Analysis

All statistical analyses were performed using IBM SPSS Statistics 21 (IBM Corp., Armonk, NY, USA). Data were analyzed as a completely randomized design using one-way ANOVA, with the dietary treatment as the fixed effect. The replicate cage was considered the experimental unit for growth performance and excreta measurements. For sampled traits, including serum indices, intestinal morphology, gene expression, TEM, and meat quality measurements, each sampled bird was selected from a different replicate cage; therefore, the sampled bird represented its corresponding cage for statistical analysis. For carcass traits at day 43, two birds were sampled from each cage and their values were averaged within the cage before statistical analysis. For relative gene expression data, statistical analyses were performed on the ∆*Ct* values (or ∆∆*Ct* values) prior to conversion to 2^−∆∆Ct^ fold changes to satisfy the assumptions of a normal distribution and homogeneity of variance. Data are presented as treatment means with pooled SEMs. Normality and homogeneity of variance were checked before analysis. When the assumption of homogeneity of variance was satisfied, data were analyzed using one-way ANOVA, followed by Duncan’s multiple-range test for multiple comparisons. When variances were unequal, Welch’s ANOVA was used, followed by the Games–Howell test for multiple comparisons. Statistical significance was declared at *p* < 0.05, and 0.05 ≤ *p* < 0.10 was considered a tendency.

## 3. Results

### 3.1. Growth Performance

As shown in Table 2, the dietary treatments had no significant effects on body weight (BW), the average daily gain (ADG), the average daily feed intake (ADFI), or the feed conversion ratio (FCR) during any individual feeding phase or over the overall 43-day experimental period (*p* > 0.05).

### 3.2. Carcass Traits

The dressed percentage (DP) and eviscerated yield (EP) were significantly affected by the dietary treatments (Table 3; *p* < 0.01). Specifically, birds fed the PNA-FM, PNA-PM, and PNA-NM diets exhibited higher DP and EP values compared with the CON group. Furthermore, DP was significantly higher in the PNA-FM group than in the PNA-NM group (*p* < 0.01). No significant treatment differences were observed for the half-eviscerated yield with giblets (HEP), abdominal fat yield (AEP), breast muscle yield (BMP), or leg muscle yield (LMP) (*p* > 0.05).

### 3.3. Meat Quality

The meat quality parameters at day 42 are summarized in Table 4. The muscle pH was significantly elevated in the PNA-PM and PNA-NM groups compared to the CON and PNA-FM groups (*p* < 0.01). Drip loss increased in all PNA-supplemented groups (PNA-FM, PNA-PM, and PNA-NM) relative to the CON group (*p* < 0.05), while the shear force was only significantly higher in the PNA-NM group compared to CON (*p* < 0.05). Histological analysis revealed a lower collagen volume fraction (CVF) in both the PNA-PM and PNA-NM groups relative to CON (*p* < 0.05). No significant treatment effects were detected for cooking loss, antioxidant indices (GSH, MDA, T-AOC), the white striping score, or the muscle fiber diameter (MFD) (*p* > 0.05).

### 3.4. Intestinal Barrier-Related Indices, Gene Expression, and Morphology

Table 5 presents the intestinal barrier-related indices at day 14. The serum FITC-d concentrations varied significantly among treatments (*p* < 0.01), peaking in the PNA-PM group (where it was higher than in both CON and PNA-FM) and also showing elevated levels in the PNA-NM group compared to CON. Serum DAO activity showed a tendency to differ among treatments (*p* = 0.081), whereas D-LA, sIgA, and COX-2 were not affected (*p* > 0.05).

Regarding ileal gene expression (Table 6), the relative expression of *ZO-1* was downregulated in the PNA-NM group compared with CON (*p* < 0.05). Additionally, the expression levels of *CHGA*, *FAAH*, and *NAPEPLD* were significantly upregulated in specific PNA-supplemented groups—most notably in PNA-FM and PNA-PM—compared to the CON group (*p* < 0.05). The expression of other measured tight junction, cytokine, and intestinal marker genes did not differ significantly among treatments (*p* > 0.05).

The ileal histomorphology results (Table 7) indicated that the villus height and villus height-to-crypt depth ratio were significantly reduced in all PNA-supplemented groups (PNA-FM, PNA-PM, and PNA-NM) compared to CON (*p* < 0.01). The crypt depth was significantly deeper in the PNA-NM group than in all other treatments (*p* < 0.01). Representative morphometric images are shown in Figure 1. Furthermore, the qualitative assessment of TEM images at day 22 suggested that epithelial junctions appeared closer, with reduced intercellular spacing, in the PNA-containing treatments compared with CON (Figure 2). Because this assessment was qualitative and performed at a later time point than the day 14 barrier-related measurements, the TEM observations were interpreted descriptively.

### 3.5. Lesion Incidence and Excreta Nutrient Composition

The overall incidence of typical clinical lesions from days 1 to 42 (Figure 3) did not differ significantly among treatments (*p* > 0.05). The excreta nutrient composition data are detailed in Table 8. On day 28, the ether extract content was significantly lower in the PNA-FM group compared to all other treatments (*p* < 0.05), and the phosphorus content was lower in PNA-FM than in PNA-PM and PNA-NM (*p* < 0.05). On day 14, excreta calcium was elevated in the PNA-PM group relative to the CON and PNA-NM groups (*p* < 0.05). No other measured excreta components showed significant treatment differences (*p* > 0.05). These results describe changes in excreta nutrient composition and were not used to calculate apparent nutrient digestibility or nutrient retention.

## 4. Discussion

### 4.1. Matrix Credit Strategy and Growth Performance

The present study evaluated broiler responses to practical PNA-containing formulation strategies rather than to a full factorial combination of PNA supplementation and nutrient matrix levels. Accordingly, the treatment effects were discussed as responses to complete PNA-containing dietary formulation strategies rather than as independent PNA effects, independent nutrient matrix effects, or formal PNA × nutrient matrix interactions. Therefore, the observed responses should be interpreted as the combined outcomes of PNA inclusion and the corresponding dietary nutrient background. Under these conditions, no significant differences in growth performance were observed among dietary treatments, indicating that the matrix credit strategies evaluated here did not compromise body weight gain, feed intake, or feed conversion under the present management conditions. This finding is consistent with previous reports showing that essential oil- or phytogenic-based additives can be associated with maintained or improved broiler performance and nutrient-related responses under certain dietary conditions [6,7], particularly when the nutrient density is reduced or intestinal challenge conditions are present [8,16]. However, responses to phytogenic products are not uniform and may depend on the additive composition, the dietary matrix, environmental pressure, and the physiological status of the birds [10].

The absence of a growth performance penalty should be interpreted in relation to the magnitude of the nutrient adjustments. In the PNA-FM diet, the reductions in ether extract, calcium, phosphorus, and selected indispensable amino acids were intended to reflect commercial formulation margins rather than severe nutrient restriction. Such formulation changes may alter the supply of lipid-derived energy, mineral availability, and amino acid balance, all of which are relevant to growth, tissue accretion, and intestinal metabolism in broilers [11,12,13]. The maintenance of growth performance therefore suggests that the tested matrix credits remained within a range that the birds could physiologically accommodate under the present conditions. Nevertheless, this result should not be extrapolated to larger nutrient reductions or to birds exposed to stronger sanitary or environmental stress, because nutrient responses may vary with diet composition, ingredient quality, and challenge status [14,15].

### 4.2. Carcass Traits and Meat Quality

Although growth performance was unaffected, this finding does not preclude changes in carcass yield traits, because the dressed percentage and eviscerated yield are proportional traits calculated relative to the live body weight. Therefore, the higher dressed percentages and eviscerated yields observed in the PNA-containing treatments may indicate that carcass-level responses were modified by the complete formulation strategies, rather than indicating that overall body weight gain was increased. However, the absence of significant changes in breast and leg muscle yields suggests that the response was not expressed uniformly across all carcass components. This pattern may reflect differences in non-muscle carcass components, digestive tract content, visceral development, or tissue partitioning rather than a direct increase in muscle deposition. Previous studies have shown that dietary energy density and phytogenic additives can influence carcass traits in broilers [14,36,37], but the direction and magnitude of these responses are highly dependent on the diet composition, additive type, and rearing conditions [16].

The meat quality responses further indicate that the effects of PNA-containing strategies were matrix-dependent. The increase in muscle pH in PNA-PM and PNA-NM, together with the higher drip loss and shear force in PNA-NM, suggests that PNA addition, particularly without a nutrient matrix credit, may have altered postmortem muscle characteristics. The water-holding capacity and texture are influenced by postmortem pH declines, protein–water interactions, connective tissue properties, the muscle fiber structure, and cooking conditions [28,29,38]. Therefore, the higher drip loss and shear force in PNA-NM should not be interpreted as a simple beneficial response but rather as an indication that PNA addition without nutrient rebalancing may shift meat quality characteristics in a way that differs from the effects of full or protein matrix credit strategies. The lack of parallel changes in muscle GSH, MDA, and T-AOC further indicates that the observed meat quality responses cannot be explained solely by the measured antioxidant indices. Similar variability in meat quality responses has been reported in broilers receiving essential oil-based or phytogenic additives [37], supporting the view that meat quality outcomes are jointly influenced by additive application and the surrounding nutritional context.

Together with the intestinal findings, these meat quality responses suggest that the dietary matrix and PNA active components may have influenced nutrient partitioning, postmortem muscle properties, and epithelial adaptation through partially connected physiological pathways. However, because muscle antioxidant indices were not significantly altered, the changes in drip loss and shear force are more likely to reflect differences in postmortem muscle characteristics and water-holding capacity than a direct antioxidant-mediated effect.

### 4.3. Intestinal Barrier-Related Responses

The intestinal results showed a time-dependent and non-linear response pattern. At day 14, PNA-NM increased the serum FITC-d concentration, tended to increase DAO activity, and reduced *ZO-1* expression, and all PNA-containing treatments reduced the villus height and the villus height-to-crypt depth ratio. These responses suggest that the early intestinal effect of PNA-containing formulation strategies was not simply protective but may reflect transient epithelial adjustment or barrier-related remodeling. FITC-d and DAO are commonly used as indicators related to paracellular permeability and mucosal barrier status [32,33], whereas tight junction gene expression provides molecular information about barrier regulation but should be interpreted together with the intestinal morphology, immune-related indicators, and the sampling time [34,39]. Therefore, the combination of increased permeability-related markers, reduced *ZO-1* expression, and an altered villus morphology indicates an early epithelial response rather than direct evidence of improved barrier function.

The subsequent TEM observations at day 22 suggested closer epithelial junctions and reduced intercellular spacing in PNA-containing treatments. Therefore, the day 14 permeability-related changes and the day 22 TEM observations should not be interpreted as contradictory findings. Instead, they may represent different stages of a time-dependent epithelial response, with early barrier-related adjustment followed by later ultrastructural remodeling. Such a temporal pattern is biologically plausible because intestinal barrier function is dynamic and can respond rapidly to luminal substrates, microbial signals, nutrient supply, and local inflammatory or oxidative conditions [30,31,40,41]. However, because the TEM assessment was qualitative and was performed at a different time point from the FITC-d, DAO, gene expression, and morphology measurements, these findings should be considered supportive rather than definitive evidence of improved barrier function.

The gene expression responses further support the view that the intestinal response is complex and should not be interpreted as a uniformly beneficial effect. The downregulation of *ZO-1* in PNA-NM was consistent with the higher serum FITC-d concentration in this group, suggesting that the no-matrix-credit strategy may have induced a more evident early barrier-related adjustment. In contrast, the significant upregulation of *CHGA*, *FAAH*, and *NAPEPLD* in selected PNA-containing treatments may indicate changes in epithelial–endocrine or lipid-mediated signaling during early intestinal adaptation. CHGA has been reported as a biomarker associated with neuroendocrine and inflammatory responses under systemic stress conditions [42], whereas *FAAH* is a key enzyme involved in fatty acid amide metabolism and the termination of endocannabinoid signaling [43,44]. *NAPEPLD* is involved in the biosynthesis of N-acylethanolamines, which are related to lipid-mediated signaling pathways [43]. Nevertheless, these gene expression changes do not establish a causal endocannabinoid mechanism. They should instead be interpreted as molecular evidence that the PNA-containing formulation strategies altered epithelial signaling during early intestinal adaptation.

The dietary matrix may partly explain the divergent intestinal responses observed among treatments. In the PNA-FM formulation, the moderate reductions in ether extract, calcium, phosphorus, and selected indispensable amino acids could have changed the luminal substrate availability, mineral dynamics, and digestive conditions. In the PNA-PM formulation, the adjustment of crude protein and the standardized ileal digestible amino acid balance may have influenced nitrogen metabolism, mucosal turnover, and the availability of amino acids required for epithelial maintenance. Under these nutrient backgrounds, the essential oils, spice-derived compounds, and saponins in the PNA may have contributed to changes in microbial pressure, epithelial signaling, and local inflammatory or oxidative status [3,4,5,8,9,25,26]. However, because the experimental design did not include PNA-free full matrix or protein matrix control diets, these intestinal responses should be interpreted as the effects of complete dietary formulation strategies rather than independent effects of the PNA or formal PNA × nutrient matrix interactions.

### 4.4. Excreta Nutrient Composition

The excreta data provide supportive evidence that nutrient-related responses differed among the formulation strategies. At day 28, the ether extract concentration was lower in PNA-FM than in the other treatments, while phosphorus was lower in PNA-FM than in PNA-PM and PNA-NM. At day 14, the calcium concentration was higher in PNA-PM than in CON and PNA-NM. These changes are consistent with the idea that matrix credit formulation alters nutrient flow through the digestive tract, because dietary formulation strategies that modify lipid and mineral supply can influence nutrient digestibility and Ca–P responses in broilers [45,46]. The lower excreta ether extract concentration in PNA-FM may reflect changes in lipid digestion, lipid excretion, or dietary substrate supply, but it cannot by itself prove improved fat digestibility, because apparent digestibility or nutrient retention analysis requires marker-based, ileal, or total excreta collection approaches rather than excreta nutrient concentrations alone [45,47]. Similarly, the changes in excreta calcium and phosphorus may reflect differences in dietary mineral input, intestinal mineral handling, or excretion patterns rather than direct improvements in mineral utilization [46,48]. These excreta responses may be linked to the different nutrient backgrounds created by the matrix credit strategies. Changes in ether extract, calcium, phosphorus, and amino acid balance could alter luminal nutrient flow, digestive substrate availability, and epithelial nutrient handling, which may partly explain the treatment-dependent changes in excreta ether extract, calcium, and phosphorus. However, because the present study measured the excreta nutrient composition rather than apparent digestibility or nutrient retention, these findings should be interpreted as compositional evidence of altered nutrient-related responses rather than direct evidence of improved nutrient utilization.

Phytogenic additives and essential oil products have been reported to affect nutrient digestibility, energy use, the intestinal morphology, and microbial ecology in broilers [6,7,8,9,17]. These effects could theoretically contribute to altered nutrient excretion by modifying digestive enzyme activity, microbial fermentation, intestinal transit, or epithelial absorptive capacity. However, the present study measured the excreta composition rather than apparent nutrient digestibility, retention, or total nutrient balance. Therefore, the excreta results should be interpreted as compositional evidence of altered nutrient-related responses rather than direct proof of improved nutrient utilization. Future studies using factorial designs, digestibility assays, microbiota profiling, digestive enzyme measurements, and time-course barrier function assessments are needed to distinguish the independent effects of PNAs from those of nutrient matrix credits and to clarify the mechanisms underlying these matrix-dependent responses.

## 5. Conclusions

Under the conditions of this study, PNA-containing formulation strategies did not compromise broiler growth performance but were associated with changes in carcass traits, meat quality traits, intestinal barrier-related responses, and excreta nutrient composition. Crucially, the magnitude and nature of these responses varied according to the specific nutrient matrix credit applied. For instance, while all PNA-supplemented diets (PNA-FM, PNA-PM, and PNA-NM) uniformly increased the dressed percentage and eviscerated yield, omitting the matrix credit (PNA-NM) produced more pronounced shifts in meat quality parameters and intestinal barrier-related markers relative to the CON group. Furthermore, the intestinal data highlighted a dynamic, time-dependent adaptation, characterized by transient permeability shifts at day 14 followed by the subsequent ultrastructural tightening of epithelial junctions at day 22. Ultimately, these findings underscore the biological importance of evaluating functional additives within the context of their practical baseline formulations, warranting future factorial studies to uncouple and precisely define the independent and interactive effects of PNAs and specific nutrient matrix credits. Accordingly, these findings should be interpreted as responses to complete PNA-containing formulation strategies rather than as formal evidence of independent PNA effects or PNA × nutrient matrix interactions.

## Figures and Tables

**Figure 1:**
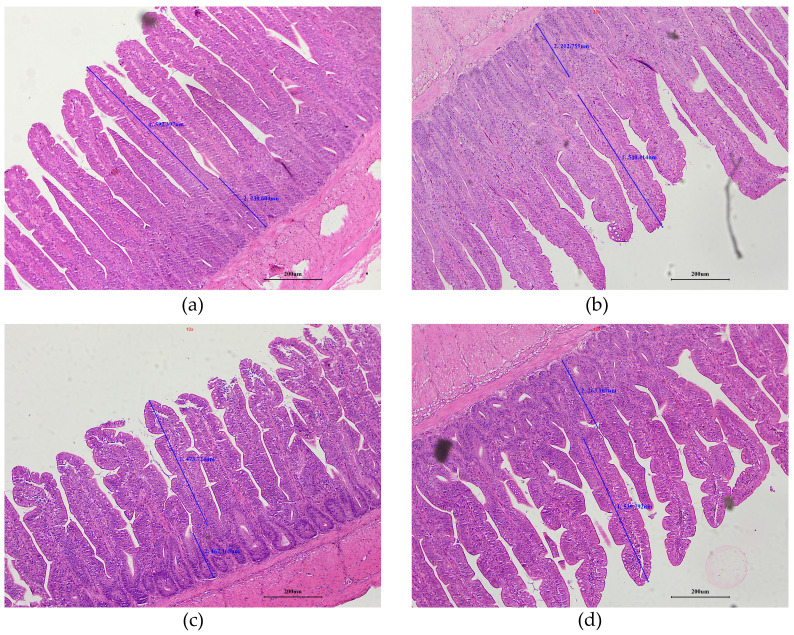
Representative morphometric measurements in the ilea of broiler chickens fed the experimental diets on day 14. (**a**) CON, control diet without PNA; (**b**) PNA-FM, PNA with a full nutrient matrix credit; (**c**) PNA-PM, PNA with a protein matrix credit; (**d**) PNA-NM, PNA without a matrix credit.

**Figure 2:**
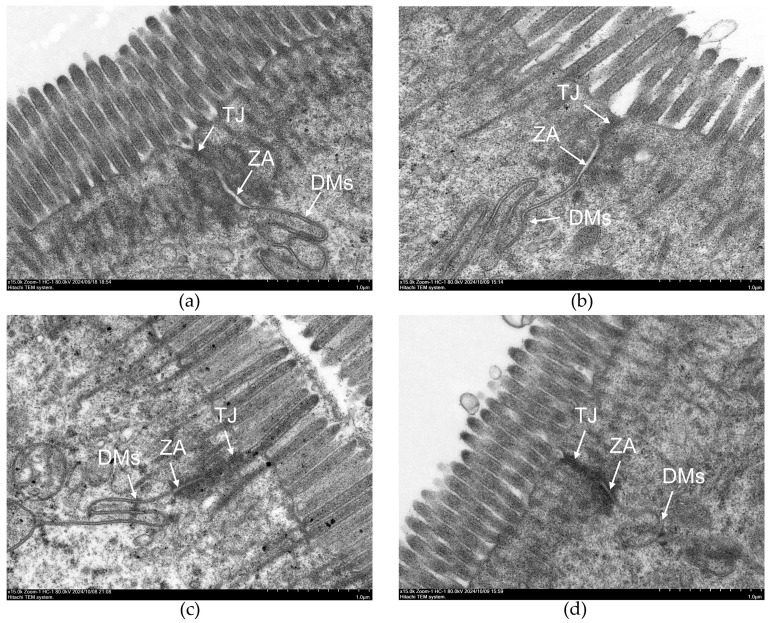
Transmission electron microscopy images of the ilea of broiler chickens fed the experimental diets on day 22. TJ = tight junction; ZA = zonula adherens; DMs = desmosomes. (**a**) CON, control diet without PNA; (**b**) PNA-FM, PNA with a full nutrient matrix credit; (**c**) PNA-PM, PNA with a protein matrix credit; (**d**) PNA-NM, PNA without a matrix credit (Hitachi, Chiyoda, Japan).

**Figure 3:**
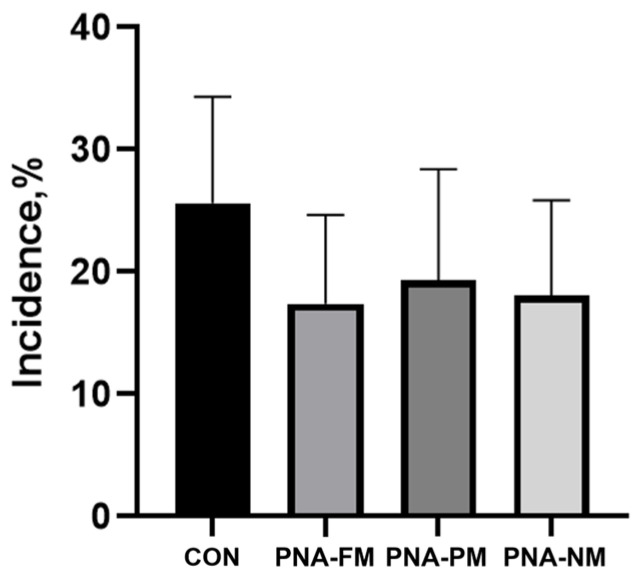
Incidence of typical lesions in broilers from days 1 to 42 in the experimental treatments.

**Table 1:** Composition and nutrient levels of the basal diets.

Item	1–14 Days	15–28 Days	29–43 Days	Item	1–14 Days	15–28 Days	29–43 Days
Ingredient, %				Nutrient levels			
Corn	44.805	41.410	37.638	Gross energy, Mcal/kg	4.244	4.306	4.519
Soybean Meal	35.200	30.530	28.370	Metabolizable energy, Mcal/kg	2.809	2.933	3.143
Wheat Flour < 4% NDF	6.000	12.000	15.000	Crude protein, %	24.699	23.325	22.115
Soybean Oil	4.160	5.520	7.620	Total calcium, %	0.791	0.567	0.479
Cottonseed Kernel Protein No Gossypol	3.500	4.000	4.500	Total phosphorus, %	0.670	0.551	0.510
Corn Gluten Meal 60% Protein	2.500	3.500	4.000	Lysine, %	1.635	1.485	1.300
Phosphate Dicalcium	1.330	0.760	0.590	Methionine, %	0.640	0.655	0.595
Calcium Carbonate 38%	0.910	0.700	0.580	Threonine, %	1.155	1.010	0.950
Salt	0.330	0.350	0.325	Tryptophan, %	0.290	0.273	0.269
AA-L-Lysine HCL 78.8%	0.310	0.350	0.400				
AA-DL Methionine-Dry 99%	0.330	0.310	0.330				
AA-Threonine	0.180	0.140	0.155				
L-Valine	0.050	0.054	0.076				
Choline Chloride-60	0.120	0.100	0.100				
Heat-Resistant Phytase	0.015	0.015	0.015				
AA-L-Isoleucine	-	0.004	0.019				
AA-Arginine 98.5%	-	-	0.023				
AA-Tryptophan 100%	-	-	0.004				
Premix ^1^	0.260	0.255	0.255				
Total	100.000	100.000	100.000				

^1^ Premix provided the following per kilogram of complete diet: vitamin A, 9000 IU; vitamin D3, 3000 IU; vitamin E, 30 IU; vitamin K3, 2.5 mg; vitamin B1, 2 mg; vitamin B2, 6 mg; vitamin B12, 0.025 mg; niacin, 50 mg; pantothenic acid, 12 mg; folic acid, 1.25 mg; biotin, 0.0325 mg; copper, 8 mg (CuSO_4_·5H_2_O); zinc, 80 mg (ZnSO_4_·H_2_O); iron, 60 mg (FeSO_4_); manganese, 80 mg (MnSO_4_·H_2_O); iodine, 0.35 mg (KI); selenium, 0.3 mg (Na_2_SeO_3_); Rovabio^®^ Advance (Adisseo, Antony, France), 300 mg.

**Table 2:** Effects of dietary treatments on growth performance of broilers ^1^.

Item	Days of Age	Group ^2^				SEM	*p*-Value
		CON	PNA-FM	PNA-PM	PNA-NM		
BW, g	1	42.47	42.01	42.02	42.13	0.107	0.392
	7	156.68	157.72	149.16	156.15	1.824	0.317
	13	369.01	371.04	356.67	372.65	3.367	0.329
	21	896.34	911.08	894.25	918.18	5.970	0.452
	28	1543.08	1555.19	1553.86	1608.69	8.375	0.056
	35	2260.82	2301.91	2301.98	2338.05	13.709	0.406
	41	2773.78	2880.13	2858.58	2916.71	18.978	0.056
ADG, g/bird	1–7	16.31	16.53	15.31	16.28	0.256	0.319
	8–13	34.76	35.55	35.55	35.87	0.442	0.851
	14–21	67.59	67.56	67.23	68.80	0.497	0.748
	22–28	93.34	92.18	94.27	98.03	0.900	0.103
	29–35	106.19	106.68	107.13	106.52	0.995	0.991
	36–41	90.56	96.72	93.02	96.21	1.236	0.270
	1–41	67.20	69.22	68.70	70.11	0.437	0.155
ADFI, g/bird	1–7	16.54	17.28	16.19	17.51	0.286	0.342
	8–13	42.26	43.11	41.56	40.76	0.467	0.307
	14–21	81.01	84.18	82.08	83.16	1.002	0.721
	22–28	131.57	129.88	132.14	135.47	1.070	0.345
	29–35	166.81	164.00	162.85	163.13	1.676	0.855
	36–41	167.51	175.81	178.11	180.57	2.882	0.476
	1–41	99.87	101.83	100.06	102.52	0.714	0.506
FCR, g/g	1–7	1.02	1.05	1.06	1.06	0.008	0.092
	8–13	1.19	1.21	1.20	1.13	0.011	0.051
	14–21	1.20	1.25	1.22	1.24	0.014	0.653
	22–28	1.42	1.43	1.38	1.42	0.014	0.664
	29–35	1.53	1.54	1.52	1.55	0.008	0.674
	36–41	1.94	1.87	1.93	1.81	0.033	0.478
	1–41	1.48	1.48	1.46	1.45	0.007	0.258

BW = body weight; ADG = average daily gain; ADFI = average daily feed intake; FCR = feed conversion ratio. ^1^ Values of means represent 8 replicated pens per treatment. ^2^ CON = control diet without PNA; PNA-FM = PNA with a full nutrient matrix credit; PNA-PM = PNA with a protein matrix credit; PNA-NM = PNA without a matrix credit.

**Table 3:** Effects of dietary treatments on carcass traits of broilers ^1^ (43 day).

Item	Group ^2^				SEM	*p*-Value
	CON	PNA-FM	PNA-PM	PNA-NM		
DP, %	92.97 ^c^	94.30 ^a^	93.95 ^ab^	93.56 ^b^	0.111	<0.001
HEP, %	86.80	88.05	87.48	87.31	0.188	0.128
EP, %	74.32 ^b^	76.40 ^a^	75.69 ^a^	76.26 ^a^	0.233	0.003
AEP, %	1.47	1.46	1.55	1.44	0.466	0.880
BMP, %	29.25	29.09	29.49	29.07	0.366	0.978
LMP, %	20.04	21.11	20.55	20.49	0.196	0.286

DP = dressed percentage, which was calculated as the weight of the carcass after exsanguination, the removal of feathers, and the removal of the horny layers of the feet, toes, and beak, divided by the live weight before slaughter × 100; HEP = percentage of half-eviscerated yield with giblets; EP = percentage of eviscerated yield; AEP = percentage of abdominal fat yield; BMP = percentage of breast muscle yield; LMP = percentage of leg muscle yield. ^1^ Values of means represent 8 replicated pens per treatment. ^2^ CON = control diet without PNA; PNA-FM = PNA with a full nutrient matrix credit; PNA-PM = PNA with a protein matrix credit; PNA-NM = PNA without a matrix credit. ^a–c^ Within a row, means with different superscripts differ significantly (*p* < 0.05).

**Table 4:** Effects of dietary treatments on meat quality of broilers ^1^ (42 day).

Item	Group ^2^				SEM	*p*-Value
	CON	PNA-FM	PNA-PM	PNA-NM		
pH	6.46 ^b^	6.71 ^b^	6.69 ^a^	6.78 ^a^	0.034	0.001
DL, %	3.15 ^c^	4.17 ^b^	5.70 ^a^	5.58 ^a^	0.251	<0.001
CL, %	24.47	22.88	22.95	25.50	0.710	0.519
GSH, μmol/g protein	44.79	39.50	40.49	42.70	2.593	0.900
MDA, nmol/mg protein	2.10	2.27	2.44	2.68	0.102	0.237
T-AOC, mmol/g	0.57	0.52	0.56	0.56	0.035	0.965
WSS	1.30	0.92	1.13	1.30	0.082	0.262
CVF, %	2.31 ^a^	1.40 ^ab^	0.88 ^b^	0.98 ^b^	0.186	0.016
MFD, μm	86.47	71.11	79.81	75.17	2.385	0.114
SF, N	64.79 ^b^	71.44 ^ab^	57.33 ^b^	87.08 ^a^	3.596	0.015

^1^ Values of means represent 8 replicated pens per treatment. ^2^ CON = control diet without PNA; PNA-FM = PNA with a full nutrient matrix credit; PNA-PM = PNA with a protein matrix credit; PNA-NM = PNA without a matrix credit. ^a–c^ Within a row, means with different superscripts differ significantly (*p* < 0.05). DL = drip loss; CL = cooking loss; GSH = glutathione; MDA = malondialdehyde; T-AOC = total antioxidant capacity; WSS = white striping score; CVF = collagen volume fraction; MFD = muscle fiber diameter; SF = shear force.

**Table 5:** Effects of dietary treatments on intestinal barrier-related indices of broilers ^1^ (14 day).

Item	Group ^2^				SEM	*p*-Value
	CON	PNA-FM	PNA-PM	PNA-NM		
FITC-d, ng/mL	1.66 ^c^	1.72 ^bc^	1.78 ^a^	1.75 ^ab^	0.013	0.002
D-LA, mmol/L	2.84	2.73	2.39	2.47	0.119	0.510
DAO, U/L	33.25	84.83	79.04	44.71	8.550	0.081
sIgA, ng/mL	398.73	364.59	367.04	345.01	13.99	0.623
COX-2, ng/mL	38.79	34.71	33.07	34.22	0.877	0.122

^1^ Values of means represent 6 replicated pens per treatment. ^2^ CON = control diet without PNA; PNA-FM = PNA with a full nutrient matrix credit; PNA-PM = PNA with a protein matrix credit; PNA-NM = PNA without a matrix credit. ^a–c^ Within a row, means with different superscripts differ significantly (*p* < 0.05). FITC-d = fluorescein isothiocyanate–dextran; D-LA = D-lactic acid; DAO = diamine oxidase activity; sIgA = secretory immunoglobulin A; COX-2 = cyclooxygenase-2.

**Table 6:** Effects of dietary treatments on ileal gene expression of broilers ^1^ (14 day).

Item		Group ^2^				SEM	*p*-Value
		CON ^3^	PNA-FM	PNA-PM	PNA-NM		
Tight junctions	*CLDN-1*	1.00	0.55	0.69	0.32	0.123	0.275
	*CLDN-2*	1.00	0.99	0.95	0.84	0.052	0.733
	*OCLN*	1.00	0.86	0.82	0.59	0.058	0.083
	*ZO-1*	1.00 ^a^	0.77 ^ab^	0.78 ^ab^	0.57 ^b^	0.047	0.007
	*ZO-2*	1.00	1.04	1.10	0.85	0.043	0.206
Cytokines	*IL-1β*	1.00	2.02	1.38	0.60	0.233	0.165
	*IL-10*	1.00	1.31	0.92	0.54	0.154	0.384
	*IL-22*	1.00	1.22	0.76	0.43	0.138	0.216
	*IL-15*	1.00	0.90	1.12	0.88	0.053	0.352
Intestinal marker genes	*MUC-2*	1.00	0.78	0.77	0.60	0.056	0.078
	*LGR5*	1.00	0.67	0.66	0.53	0.073	0.133
	*SOX9*	1.00	1.53	1.62	1.09	0.213	0.689
	*CHGA*	1.00 ^b^	1.96 ^a^	1.88 ^a^	1.33 ^b^	0.118	0.003
	*LYZ*	1.00	1.15	1.69	1.12	0.156	0.423
	*FAAH*	1.00 ^b^	2.43 ^a^	2.35 ^a^	1.59 ^ab^	0.201	0.024
	*CNR1*	1.00	0.75	0.73	0.63	0.067	0.258
	*NAPEPLD*	1.00 ^c^	2.41 ^ab^	3.35 ^a^	1.91 ^bc^	0.236	0.001

^1^ Values of means represent 6 replicated pens per treatment. Primer sequences are shown in Table A1. ^2^ CON = control diet without PNA; PNA-FM = PNA with a full nutrient matrix credit; PNA-PM = PNA with a protein matrix credit; PNA-NM = PNA without a matrix credit. ^3^ CON was used as the calibrator group for the calculation of relative gene expression using the 2^−ΔΔCt^ method and is therefore presented as 1.00. This value represents the normalized reference level and does not indicate an absence of biological variation. ^a–c^ Within a row, means with different superscripts differ significantly (*p* < 0.05).

**Table 7:** Effects of dietary treatments on ileal morphology in broilers ^1^ (14 days).

Item	Group ^2^				SEM	*p*-Value
	CON	PNA-FM	PNA-PM	PNA-NM		
VH, μm	556.54 ^a^	489.48 ^b^	469.62 ^b^	457.66 ^b^	6.729	<0.001
CD, μm	185.09 ^b^	180.26 ^b^	181.50 ^b^	218.93 ^a^	3.366	<0.001
V/C, μm/μm	3.10 ^a^	2.73 ^b^	2.63 ^b^	2.14 ^c^	0.046	<0.001

^1^ Values of means represent 6 replicated pens per treatment. ^2^ CON = control diet without PNA; PNA-FM = PNA with a full nutrient matrix credit; PNA-PM = PNA with a protein matrix credit; PNA-NM = PNA without a matrix credit. ^a–c^ Within a row, means with different superscripts differ significantly (*p* < 0.05). VH = villus height; CD = crypt depth; V/C = ratio of villus height to crypt depth.

**Table 8:** Effects of dietary treatments on excreta nutrient composition in broilers at days 14, 28, and 42 ^1^.

Item	Days of Age	Group ^2^				SEM	*p*-Value
		CON	PNA-FM	PNA-PM	PNA-NM		
Gross energy, MJ/kg	14	15.51	15.61	15.81	15.87	0.067	0.155
	28	15.20	14.91	15.02	14.92	0.086	0.608
CP, %	14	31.49	32.06	33.41	32.52	0.443	0.499
	28	40.61	49.44	48.88	42.36	1.833	0.226
CF, %	14	10.63	11.22	10.77	11.78	0.196	0.177
	28	11.34	11.23	10.63	11.62	0.243	0.588
EE, %	14	2.75	2.60	3.64	3.34	0.164	0.091
	28	3.83 ^a^	2.95 ^b^	3.83 ^a^	3.89 ^a^	0.133	0.017
Ash, %	14	9.37	9.57	9.44	9.15	0.086	0.379
	28	11.86	11.78	12.40	11.64	0.254	0.726
Ca, %	14	0.36 ^b^	0.42 ^ab^	0.49 ^a^	0.33 ^b^	0.020	0.010
	28	0.48	0.31	0.40	0.47	0.027	0.099
P, %	14	0.68	0.72	0.75	0.67	0.017	0.371
	28	0.93 ^ab^	0.78 ^b^	1.00 ^a^	1.05 ^a^	0.034	0.024
Moisture, %	14	81.15	80.86	80.63	80.56	0.161	0.659
	28	80.97	80.91	81.30	81.33	0.255	0.924
	42	79.91	80.17	80.77	83.19	0.741	0.508

^1^ Values of means represent 6 replicated pens per treatment. ^2^ CON = control diet without PNA; PNA-FM = PNA with a full nutrient matrix credit; PNA-PM = PNA with a protein matrix credit; PNA-NM = PNA without a matrix credit. ^a,b^ Within a row, means with different superscripts differ significantly (*p* < 0.05). CP = crude protein; EE = ether extract; CF = crude fiber.

## Data Availability

The original contributions presented in this study are included in the article. Further inquiries can be directed to the corresponding author.

## Abbreviations

The following abbreviations are used in this manuscript:

ADFI	Average daily feed intake
ADG	Average daily gain
AEP	Abdominal fat yield
ANOVA	Analysis of variance
AOAC	Association of Official Analytical Chemists
BMP	Breast muscle yield
BW	Body weight
cDNA	Complementary DNA
CFM	Cecal gas bubbles/microbial imbalance
CL	Cooking loss
CON	Control diet without phytogenic nutritional additive
COX-2	Cyclooxygenase-2
CS	Intestinal epithelial cell shedding
CVF	Collagen volume fraction
D-LA	D-Lactic acid
DAO	Diamine oxidase
DL	Drip loss
DP	Dressed percentage
EP	Eviscerated yield
FAAH	Fatty acid amide hydrolase
FCR	Feed conversion ratio
FITC-d	Fluorescein isothiocyanate–dextran
GIZ	Gizzard erosion
GSH	Glutathione
HEP	Half-eviscerated yield with giblets
HY	Intestinal hyperemia
IH	Intestinal hemorrhage
IT	Poor intestinal elasticity
LMP	Leg muscle yield
MC	Excessive intestinal mucus
MDA	Malondialdehyde
MFD	Muscle fiber diameter
ML	Oral lesions
NAPEPLD	N-acyl phosphatidylethanolamine phospholipase D
PNA	Phytogenic nutritional additive
PNA-FM	Phytogenic nutritional additive with full nutrient matrix credit
PNA-NM	Phytogenic nutritional additive without matrix credit
PNA-PM	Phytogenic nutritional additive with protein matrix credit
qPCR	Quantitative real-time PCR
RNA	Ribonucleic acid
SEM	Standard error of the mean
sIgA	Secretory immunoglobulin A
T-AOC	Total antioxidant capacity
TEM	Transmission electron microscopy
WC	Excessive intestinal water content
ZO-1	Zonula occludens-1

## Appendix A

**Table A1:** Primer sequences of genes detected by quantitative real-time PCR.

Target Gene	Primer Sequence (5′ → 3′)	GenBank Accession Number
*β-Actin*	F: CAACACAGTGCTGTCTGGTGGTAC	NM_205518.2
	R: CTCCTGCTTGCTGATCCACATCTG	
*CLDN-1*	F: TGGAGGATGACCAGGTGAAGA	NM_001013611.2
	R: CGAGCCACTCTGTTGCCATA	
*CLDN-2*	F: CTACAGCTCCCTGCTCAACC	NM_001277622.1
	R: ACGGCTATAAGGCAAGCAAG	
*OCLN*	F: TCATCGCCTCCATCGTCTAC	NM 205128.1
	R: TCTTACTGCGCGTCTTCTGG	
*ZO-1*	F: TGTAGCCACAGCAAGAGGTG	XM_046899250.1
	R: CTGGAATGGCTCCTTGTGGT	
*ZO-2*	F: CCAGAACCCCAGAAACCTCC	XM_046934794.1
	R: TATCTGGGTGGCTGTCCGTA	
*IL-1β*	F: ACTGGGCATCAAGGGCTA	NM_204524.2
	R: GGTAGAAGATGAAGCGGGTC	
*IL-10*	F: GCTGCCAAGCCCTGTT	NM_001004414.4
	R: CCTCAAACTTCACCCTCA	
*IL-22*	F: GGTTGTCTTCTGCTGTTGTTGCTG	NM_001199614.1
	R: GCCAAGGTGTAGGTGCGATTCC	
*IL-15*	F: CCATAGGTTTCCGAGGCTTGT	NM_204571.2
	R: TCCGGCAGAGTTTTGTGTTG	
*MUC-2*	F: CGGGTGAATGGAGGACAGAG	XM_040673065.2
	R: CATACACAGTCCCTTCGGGG	
*LGR5*	F: TCAATACCTGAGCGTGCGTT	XM_040669680.2
	R: TGTGAGTGTCAAACTCTCCAGAC	
*SOX9*	F: ACGATTACACCGAGCACCAG	NM_204281.2
	R: TGAAGGTGGAGTAGAGGCCC	
*CHGA*	F: GCAAAGAGATGTGGCACGAC	XM_421330.8
	R: GCAGCCTAGAATCCCTCACC	
*LYZ*	F: GTGTGCCGCAAAATTCGAGA	NM_205281.2
	R: GCGGCTGTTGATCTGTAGGA	
*FAAH*	F: TGGGGAGAAGAAGGGTTTGC	XM_422450.7
	R: GAGGGACTGGGGAACATTGG	
*CNR1*	F: TAACCCGACCAAAGGCTGTC	XM_046914328.1
	R: ATGAACAGCAAGAGGACGCT	
*NAPEPLD*	F: ATGCAGAGATGCGGTTGTGA	NM_001030730.2
	R: CCAAGGTCCCAAGACAGACC	
